# Supplementary material for: Structural basis of RNA polymerase inhibition by viral and host factors
Source: Nat Commun. 2021 Sep 17;12:5523. doi: 10.1038/s41467-021-25666-5 (PMC8448823; doi:10.1038/s41467-021-25666-5)
Supplement: Supplementary file 1 — Supplementary Information [file 41467_2021_25666_MOESM1_ESM.pdf]

Structural basis of RNA polymerase inhibition by viral and host factors

Supplementary Methods

Saci Rpo8 modelling and reannotation

The structural model of Rpo8 has been generated in Modeller webserver based on the homology with *S. shibatae* Rpo8 and initially fitted inside the cryo-EM map of the apo-RNAP. The model was then manually edited in Coot starting from loop β5-6 to fit correctly the map followed by refinement in Phenix. Comparisons of Rpo8 between our EM structure and other archaeal RNAP structures revealed a discrepancy. In the deposited *S. solfataricus* and *S. shibatae* RNAP X-ray structures [1,2] the GLLM motif (Saci sequence YGLIV, Sso YGLLV), reported to be essential for the binding to RPB1 in yeast [3], was erroneously assigned and rendered the motif solvent exposed. In our structure, the conserved GGLLM motif is in the same position seen in all eukaryotic RPB8 at the interface with Rpo1' (Figure 1C). The improved sequence alignment shown in figure 1D predicts also that the β5-6 loop in *S. shibatae* and *S. solfataricus* should be longer than what initially published. Indeed, the electron density maps of both RNAP structures (codes 3hkz and 4ayb [4,2]) support our findings showing extra-density proximal to the loop which suggests that both loops are longer and partially disordered (Supplementary figure 3c).

Supplementary Tables

Oligo name	sequence
Cy3-TS83	[Cyanine3]-ccacccttacctccaccatatgggagatccattagagtagttaagatgaagtagttacgcctggtcattactagtactgccgg
NTS83	ccggcagtactagtaatgaccaggcgtaactacttcatcttaactactctaatggatctcccatatggtggaggtaagggtgg
RNA14	auuuagaccaggcg

Supplementary table 1. DNA and RNA sequences used to assemble the elongation scaffold.

Supplementary figures

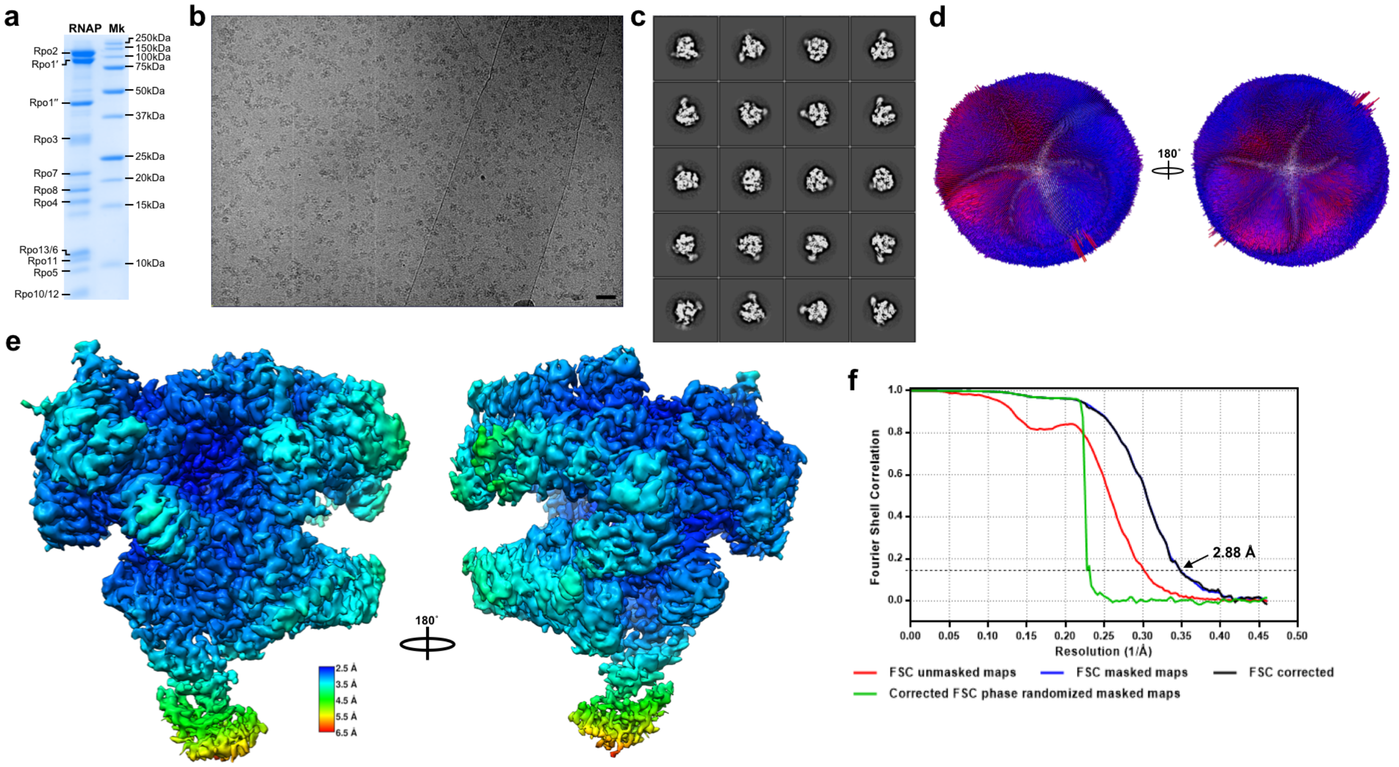

Supplementary figure 1. Cryo-EM results and map quality evaluation for the apo-RNA polymerase. a) Representative SDS-PAGE showing the RNA polymerase before crosslinking (see SOURCE DATA). b) Representative motion-corrected cryo-EM micrograph; scale bar corresponds to 300 Å. c) Selected averages of the RNA polymerase common views; box size 360.22 Å. d) Angular distribution plot illustrating the contribution of the number of particles orientations to the final em map. The plot is shown as a sphere around the em map using the same two orientations used in panel e with the over-represented angles highlighted in red. e) Local resolution variation of the RNA polymerase 3D reconstruction; map is presented in two orientations and coloured as indicated in the scale below. f) Gold standard Fourier Shell Correlation (FSC) plot obtained from post processing in Relion 3.0. The dashed line represents 0.143 cutoff which indicated a resolution of 2.88 Å. For the curve labelled 'masked', the FSC was calculated using a mask with soft edges.

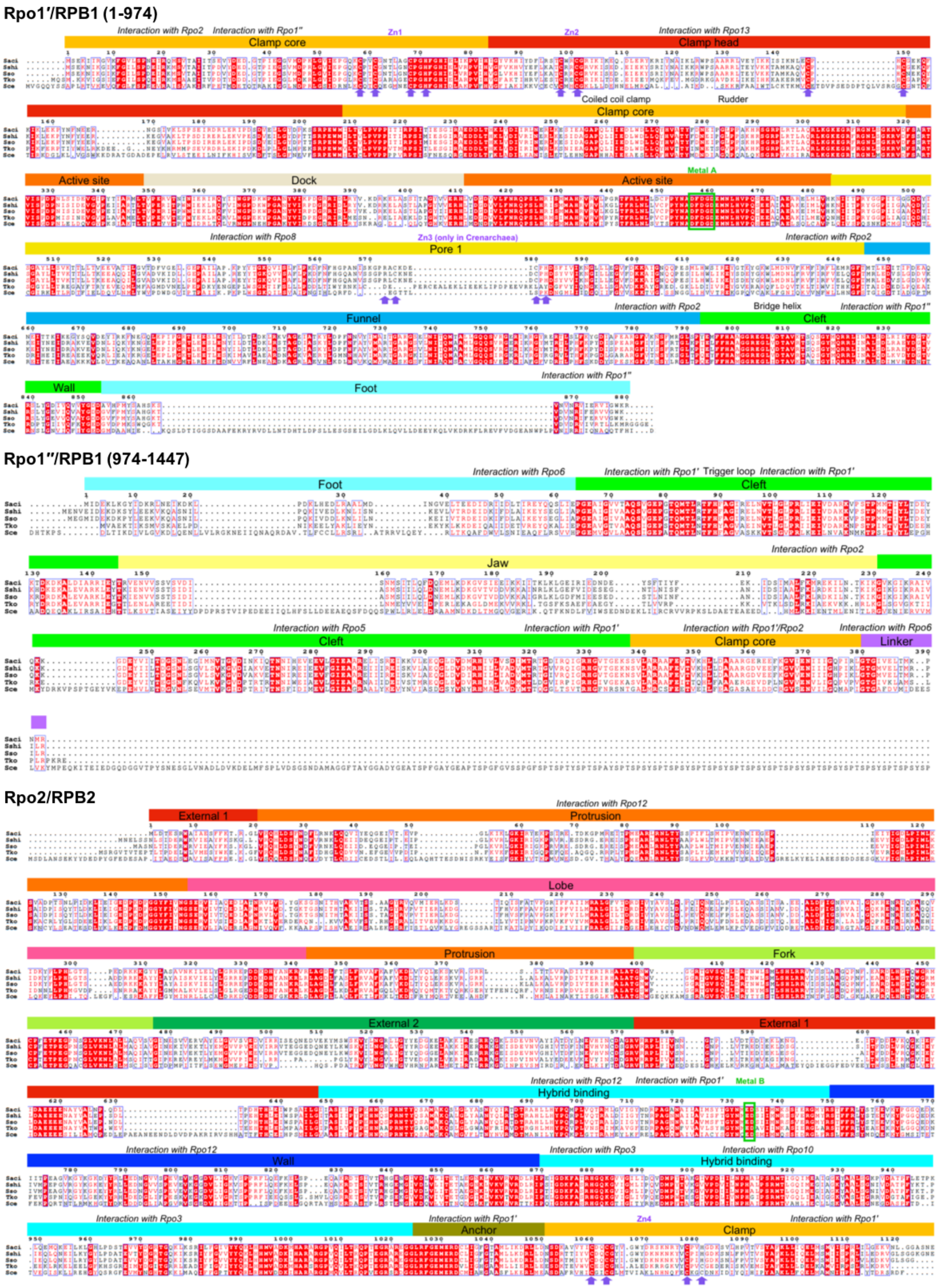

Supplementary figure 2. Structure-based alignment of RNAP subunits from all domains of life. Structure-based sequence alignment of each single RNAP subunit from *Sulfolobus acidocaldarius*, *Saccharolobus*

shibatae (pdb 4ayb [2]), *Saccharolobus solfataricus* (pdb 3hkz [4]), *Thermococcus kodakarensis* (pdb 6kf3 [5]) and the corresponding ones from *Saccharomyces cerevisiae* (pdb 6gyk [6]). The multi-sequence alignments have been manually edited to account for the unstructured loops and the domains assigned according to Cramer et al., 2001 [7]. Magnesium binding sites are highlighted within a green box and the zinc ligands with purple arrows. In archaeal Rpo1' there is a third zinc finger domain which is not conserved in yeast and in Euryarchaea.

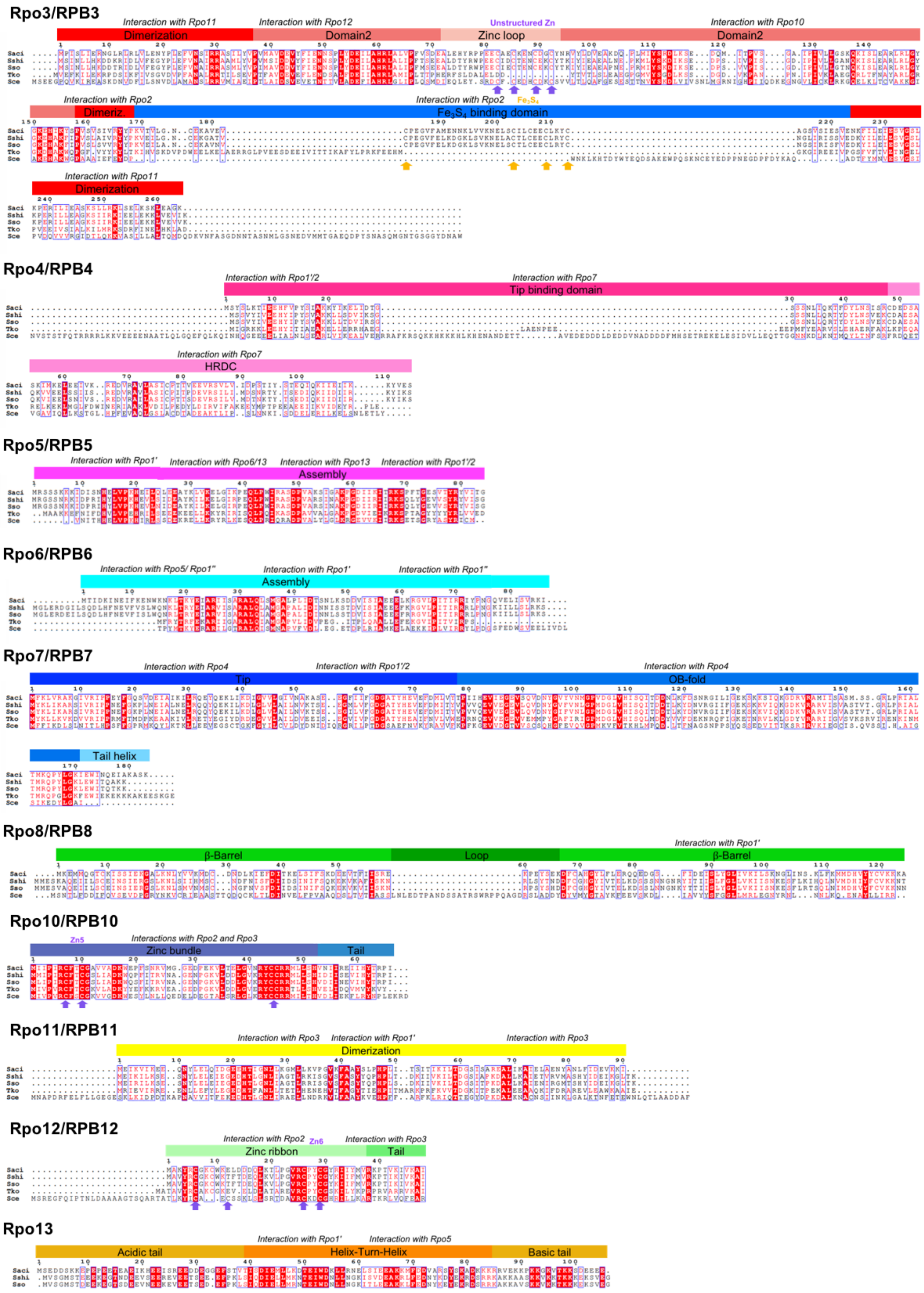
Rpo3/RPB3
Interaction with Rpo11
Interaction with Rpo12
Unstructured Zn
Interaction with Rpo10
Dimerization
Domain2
Zinc loop
Domain2
Interaction with Rpo2
Interaction with Rpo2
Dimeriz.
Fe_3S_4 binding domain
Interaction with Rpo11
Dimerization
Rpo4/RPB4
Interaction with Rpo1'/2
Interaction with Rpo7
Tip binding domain
Interaction with Rpo7
HRDC
Rpo5/RPB5
Interaction with Rpo1'
Interaction with Rpo6/13
Interaction with Rpo13
Interaction with Rpo1'/2
Assembly
Rpo6/RPB6
Interaction with Rpo5/ Rpo1"
Interaction with Rpo1'
Interaction with Rpo1"
Assembly
Rpo7/RPB7
Interaction with Rpo4
Interaction with Rpo1'/2
Interaction with Rpo4
Tip
OB-fold
Tail helix
Rpo8/RPB8
Interaction with Rpo1'
β-Barrel
Loop
β-Barrel
Rpo10/RPB10
Zn5
Interactions with Rpo2 and Rpo3
Zinc bundle
Tail
Rpo11/RPB11
Interaction with Rpo3
Interaction with Rpo1'
Interaction with Rpo3
Dimerization
Rpo12/RPB12
Interaction with Rpo2
Zn6
Interaction with Rpo3
Zinc ribbon
Tail
Rpo13
Interaction with Rpo1'
Interaction with Rpo5
Acidic tail
Helix-Turn-Helix
Basic tail

Supplementary figure 3. Structure-based alignment of RNAP subunits from all domains of life. Structure-based sequence alignment of each single RNAP subunit from *Sulfolobus acidocaldarius*, *Saccharolobus shibatae* (pdb 4ayb [2]), *Saccharolobus solfataricus* (pdb 3hkz [4]), *Thermococcus kodakarensis* (pdb 6kf3 [5]) and the corresponding ones from *Saccharomyces cerevisiae* (pdb 6gyk [6]). The multi-sequence alignments have been manually edited to account for the unstructured loops and the domains assigned according to Cramer et al., 2001 [7] and Fernandez-Tornero et al., 2013 [8]. Zinc binding sites are highlighted with purple arrows and the iron-sulfur cluster [3Fe-4S] ligands in gold arrows. The zinc ion in Saci Rpo3 is not visible because of the higher flexibility of the loop, however it has not been detected in *S. shibatae* or *S. solfataricus* although the cysteine residues are conserved. The same zinc loop is not conserved in the euryarchaeal *T. kodakaransis*. Rpo13 is a unique feature of many crenarchaeal RNA polymerases. In this figure: HRDC, Helicase RNaseD C-terminal domain; OB-fold, Oligonucleotide Binding-fold domain; tail helix stands for the C-terminal helix present only in Archaea.

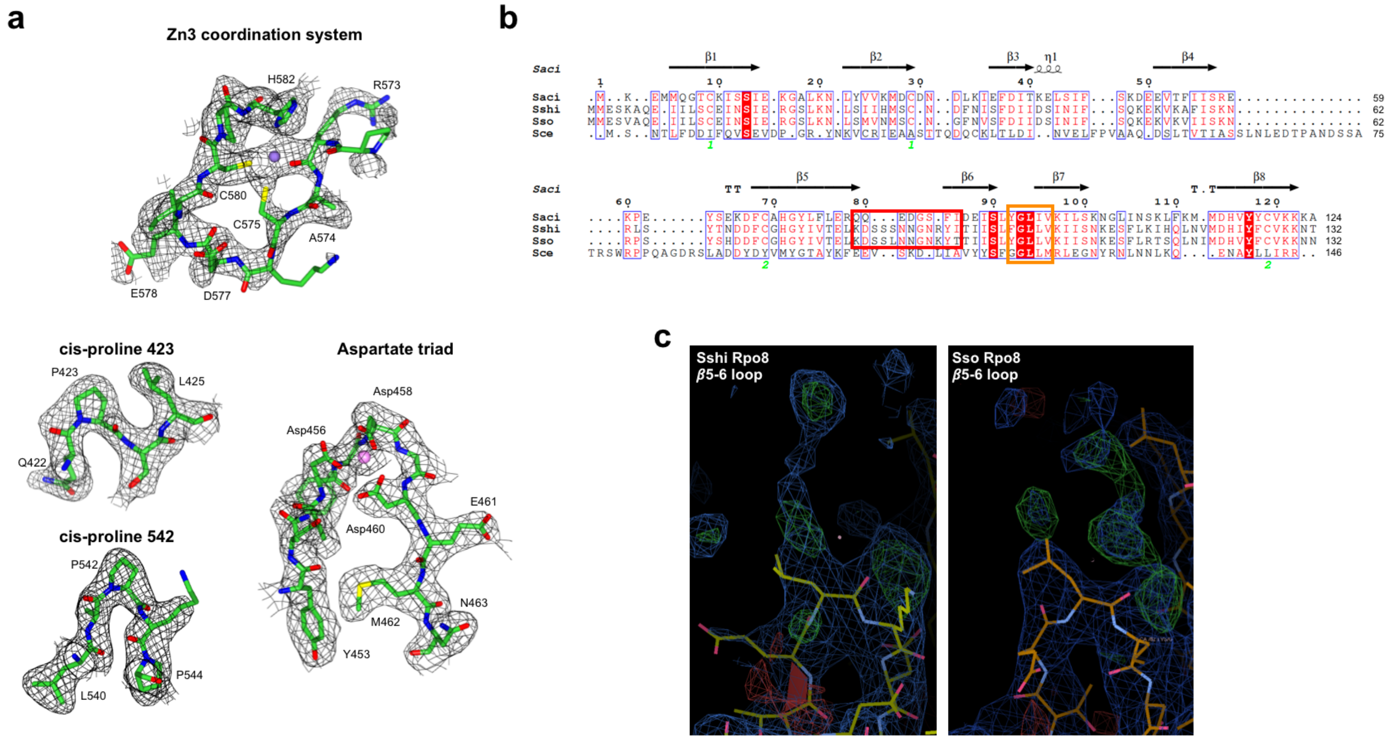

Supplementary figure 4. RNA polymerase model refinement details and validation. a) The cryo-EM map of the TFS4-bound RNAP at 2.6 Å of resolution was used to identify and assign the third zinc finger domain in Rpo1′ (Zn3) and the *cis* configuration of five prolines, four in Rpo1′ (P161/373/423/542) (two examples shown here), and one in Rpo2 (P457). Although the Zn3 system is not fully resolved (on proline 572), the map is compatible only with the presence of a zinc ion at the centre of the coordination system with the carbonyl group of Arg573 as fourth ligand. The carbonyl group is a weak ligand which might explain the local higher flexibility as well as the loss of this zinc finger domain in Euryarchaea and Eukaryotes (Supplementary figure

2). The fully coordinated aspartate triad on the catalytic loop site is also shown refined inside the same cryo-EM map. b) The improved structure-based sequence alignment of Rpo8 (Saci, Ssh pdb 4ayb, and Sso pdb 3hkz) and RPB8 (Sce pdb 1i50 [7]). The β5-6 loop is highlighted in a red box, and the GGLLM motif is highlighted with an orange box using the same colour code of figure 1c. The secondary structure annotation generated by Espript3 for the Saci Rpo8 is shown above the sequence alignments. c) Electron density map and corresponding model of the β5-6 loop from *S. shibatae* and *S. solfataricus* (pdb codes 4ayb and 3hkz, respectively [2,4]). Both density maps show a continuous extra-density on both sides of the loop (in blue the refined density and in green the positive signal) confirming that the β5-6 loop in these two species is longer than what initially established.

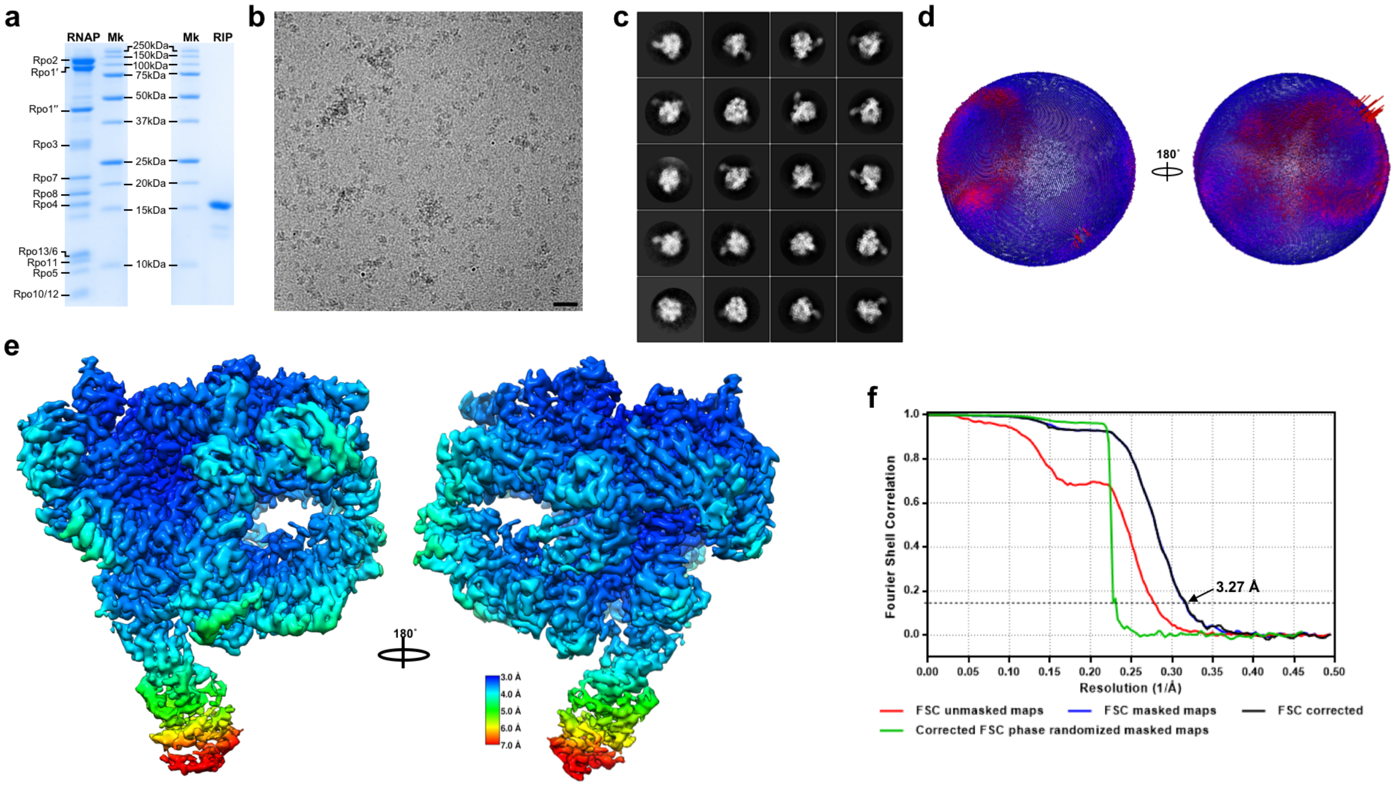

Supplementary figure 5. Cryo-EM results and map quality evaluation for the RNAP-RIP complex. a) Representative SDS-PAGE showing the RNA polymerase and RIP before crosslinking (see SOURCE DATA). b) Representative motion-corrected cryo-EM micrograph; scale bar corresponds to 300 Å. c) Selected averages of the RNA polymerase common views; box size 303 Å. d) Angular distribution plot illustrating the contribution of the number of particles orientations to the final em map. The plot is shown as a sphere around the em map using the same two orientations used in panel e with the over-represented angles highlighted in red. e) Local resolution variation of the RNA polymerase 3D reconstruction; map is presented in two orientations and coloured as indicated in the scale below. f) Gold standard Fourier Shell Correlation (FSC)

plot obtained from post processing in Relion 3.0. The dashed line represents 0.143 cutoff which indicated a resolution of 3.27 Å. For the curve labelled 'masked', the FSC was calculated using a mask with soft edges.

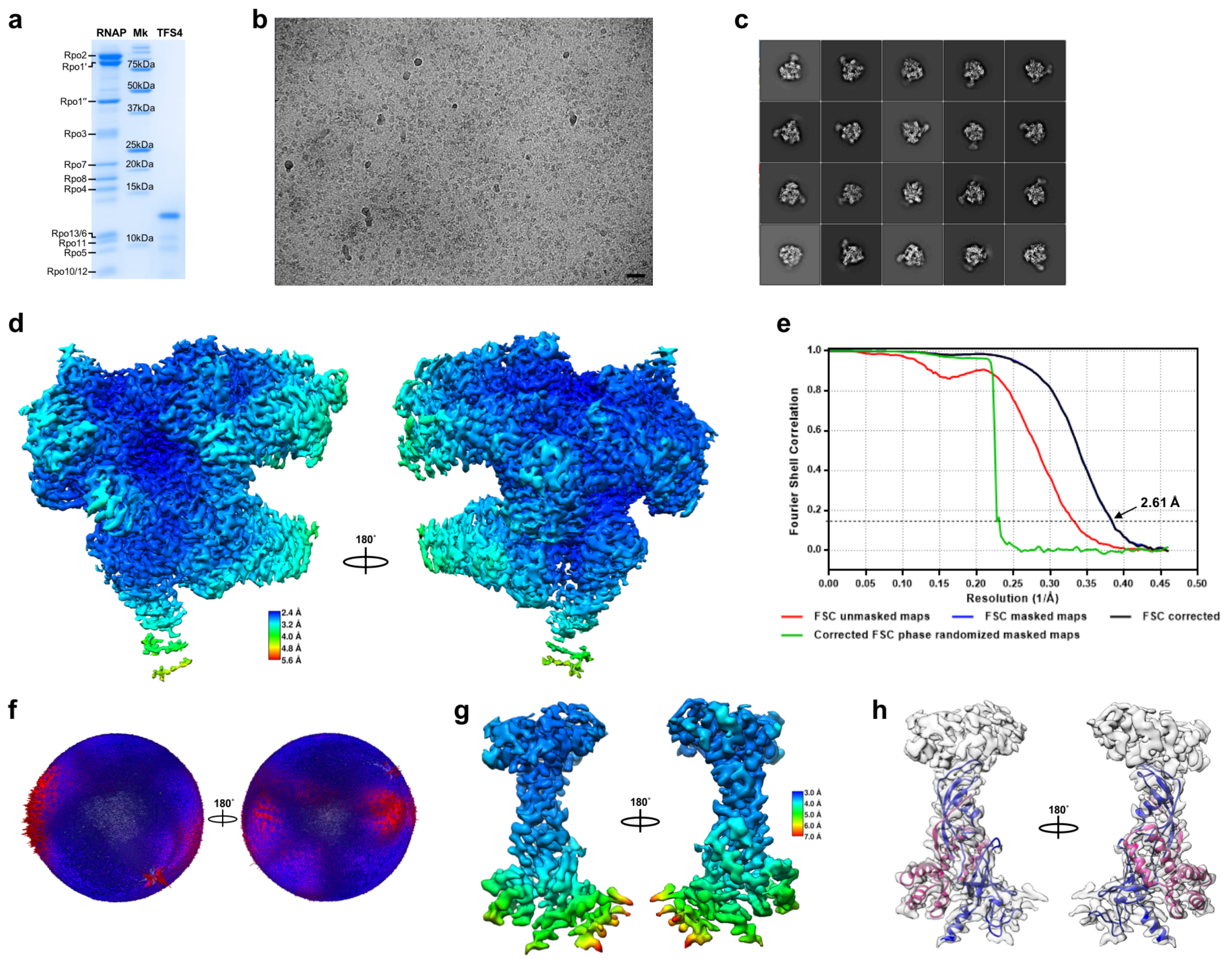

Supplementary figure 6. Cryo-EM results and map quality evaluation for the RNAP-TFS4 complex. a) Representative SDS-PAGE showing the RNA polymerase before crosslinking (see SOURCE DATA). b) Representative motion-corrected cryo-EM micrograph; scale bar corresponds to 300 Å. c) Selected averages of the RNA polymerase common views; box size 360.22 Å. d) Local resolution variation of the RNA polymerase 3D reconstruction; map is presented in two orientations and coloured as indicated in the scale below. e) Gold standard Fourier Shell Correlation (FSC) plot obtained from post processing in Relion 3.0. The dashed line represents 0.143 cutoff which indicated a resolution of 2.61 Å. For the curve labelled 'masked', the FSC was calculated using a mask with soft edges. f) Angular distribution plot illustrating the contribution of the number of particles orientations to the final em map. The plot is shown as a sphere around the em map using the same two orientations used in panel d with the over-represented angles highlighted in red. g) Local resolution variation map of the stalk map region after multi-body refinement and sharpening. h) Rpo4 (in

deep pink) and Rpo7 (in blue) subunits modelled inside the same map shown here as a semi-transparent surface.

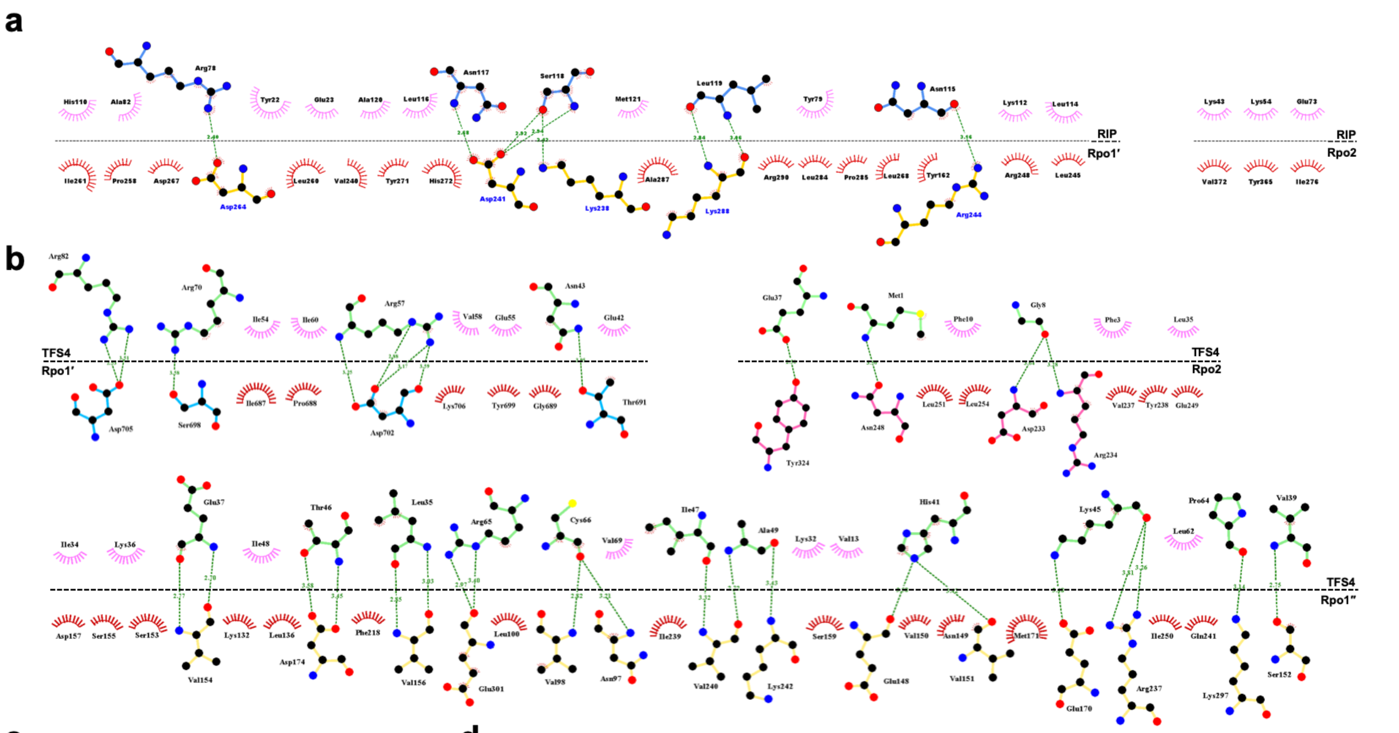

c

Subunits	Interface area	H-bonds	Salt bridges
RIP:Rpo1′	1126.87 Å	9	4
RIP:Rpo2	302.05 Å	—	2
TFS4:Rpo1′	704.41 Å	4	6
TFS4:Rpo1″	1392.11 Å	17	3
TFS4:Rpo2	506.40 Å	4	—

d

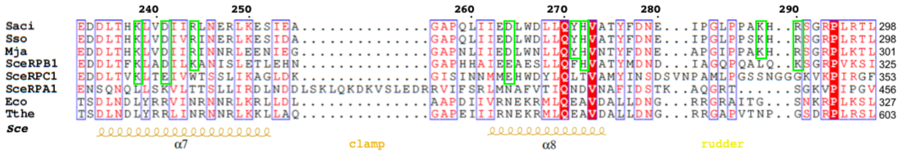

Supplementary figure 7. Interfaces analysis of RIP/RNAP and TFS4/RNAP interactions. a-b) LigPlot+ output reporting all H-bonds, salt-bridges and hydrophobic interactions. RIP and TFS4 are always reported in blue and green, respectively, the clamp/rudder residues in gold and the lobe in deep pink (a), the funnel in deep blue sky, and the jaw in yellow (b). The hydrophobic interactions are shown in magenta for RIP and TFS4 and in red for the facing subunit. c) Interface analysis carried out by PISA. d) Sequence alignment of the coiled coil clamp and rudder from different domains of life. On the bottom the secondary structure assignment for *S. cerevisiae* RPB1 is shown, and the residues directly involved in H-bond interactions with RIP are highlighted in green boxes. As result, the H-bonds are not conserved in bacteria (*E. coli* and *T. thermophilus*), and in *S. cerevisiae* RPA1 (RNAPI) and RPC1 (RNAPIII).

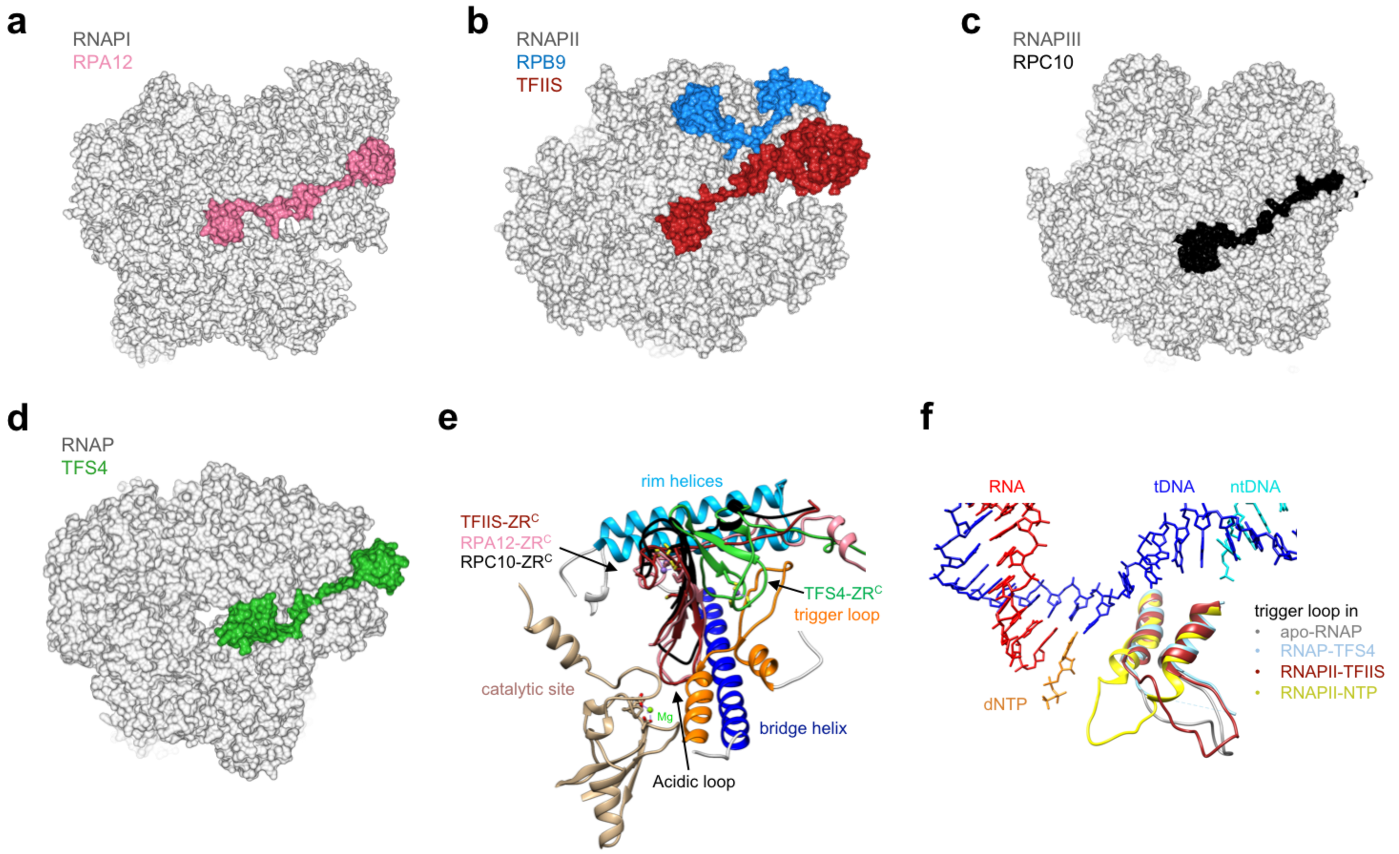

Supplementary figure 8. TFS-related proteins in RNAPI, II and III and the archaeal RNAP. Surface representations of (a) yeast RNAPI with RPA12 highlighted in pink (pdb code 6rqh [9]), (b) yeast RNAPII-TFIIS complex with RPB9 highlighted in blue and TFIIS in dark red (pdb code 5xon [10]), (c) human RNAPIII with RPC10 (homologous of yeast RPC11) in the 'inside-funnel' state highlighted in black (pdb code 7ae3 [11]), and (d) RNAP-TFS4 complex with TFS4 highlighted in green. (e) Superimposition of the ZR^C domains of TFS4 and its eukaryotic paralogues TFIIS, RPA12, and RPC10 inside the funnel. All ZR^C domains, coloured as in panels (a-d), adopt the same binding mode inside the funnel reaching the active site (in tan) through the acidic loop, interacting with both the rim helices (in sky blue) and the bridge helix (in blue) whilst not interfering with the trigger loop (in orange). In contrast, TFS4 binds at the entrance of the channel and clashes with the trigger loop with its zinc ribbon. All structures are shown in ribbons style and only RPB1 from RNAPII is shown in the image. (f) Comparison of trigger loop conformations between apo-RNAP (grey), TFS4-bound (light blue), TFIIS-locked (brown) and NTP-bound (yellow) RNAPs. Locked and closed TLs from yeast RNAPII structures (pdb code 3po3 and 2e2h, respectively [12,13]). RNA, template DNA, non-template DNA and substrate NTP are shown red, blue, cyan and orange respectively. Nucleic acids and substrate NTP were taken from 2e2h to highlight the active site. Bridge helices are omitted for clarity.

Supplementary references

1. Korkhin, Y. et al. Evolution of complex RNA polymerases: the complete archaeal RNA polymerase structure. *PLoS Biol* **7**, e1000102 (2009).
2. Wojtas, M.N., Mogni, M., Millet, O., Bell, S.D. & Abrescia, N.G. Structural and functional analyses of the interaction of archaeal RNA polymerase with DNA. *Nucleic Acids Res* **40**, 9941-52 (2012).
3. Briand, J.-F. et al. Partners of Rpb8p, a Small Subunit Shared by Yeast RNA Polymerases I, II, and III. in *Molecular and Cellular Biology* Vol. 21 6056-6065 (2001).
4. Hirata, A., Klein, B.J. & Murakami, K.S. The X-ray crystal structure of RNA polymerase from Archaea. *Nature* **451**, 851-4 (2008).
5. Jun, S.H. et al. Direct binding of TFEalpha opens DNA binding cleft of RNA polymerase. *Nat Commun* **11**, 6123 (2020).
6. Dienemann, C., Schwalb, B., Schilbach, S. & Cramer, P. Promoter Distortion and Opening in the RNA Polymerase II Cleft. *Mol Cell* **73**, 97-106 e4 (2019).
7. Cramer, P., Bushnell, D.A. & Kornberg, R.D. Structural basis of transcription: RNA polymerase II at 2.8 angstrom resolution. *Science* **292**, 1863-76 (2001).
8. Fernandez-Tornero, C. et al. Crystal structure of the 14-subunit RNA polymerase I. *Nature* **502**, 644-9 (2013).
9. Sadian, Y. et al. Molecular insight into RNA polymerase I promoter recognition and promoter melting. *Nat Commun* **10**, 5543 (2019).
10. Ehara, H. et al. Structure of the complete elongation complex of RNA polymerase II with basal factors. *Science* **357**, 921-924 (2017).
11. Girbig, M. et al. Cryo-EM structures of human RNA polymerase III in its unbound and transcribing states. *Nat Struct Mol Biol* **28**, 210-219 (2021).
12. Cheung, A.C. & Cramer, P. Structural basis of RNA polymerase II backtracking, arrest and reactivation. *Nature* **471**, 249-53 (2011).
13. Wang, D., Bushnell, D.A., Westover, K.D., Kaplan, C.D. & Kornberg, R.D. Structural basis of transcription: role of the trigger loop in substrate specificity and catalysis. *Cell* **127**, 941-54 (2006).
