# Supplementary material for: Structural basis of RNA polymerase inhibition by viral and host factors
Source: Nat Commun. 2021 Sep 17;12:5523. doi: 10.1038/s41467-021-25666-5 (PMC8448823; doi:10.1038/s41467-021-25666-5)
Supplement: Supplementary file 3 — Description of Additional Supplementary Files [file 41467_2021_25666_MOESM3_ESM.pdf]

Description of Additional Supplementary Files

Supplementary Movie 1. RNAP conformational changes induced by TFS4. The movie starts with the apo-RNAP in ribbon style with the clamp head highlighted in light blue, the jaw (Rpo1' and Rpo5) in light yellow, the trigger loop in orange, and the bridge helix in blue. The apo-RNAP structure presentation is followed by the morphing generated in Chimera using the apo-RNAP structure as initial model and the TFS4-bound structure as final model. The morphing shows the opening of the DNA-binding channel caused by the swing of the jaw downwards followed by the clamp head. After the morphing section, the apo-RNAP structure is rotated of 110˚ and 50˚ on the y and x axes, respectively. The new orientation is focused on the RNAP funnel and shows TFS4 coloured in red (ZR^N domain), rosy brown (linker), and green (ZR^C domain). The TFS4 linker overlaps with two beta strands of the upper jaw of the apo-RNAP and the ZR^C domain clashes with the trigger loop and the upper jaw leaving, however, the funnel not occluded. Finally, the movie shows again the morphing to the TFS4-bound RNAP structure to highlight the jaw swing required to allow TFS4 linker to bind to the beta strands of the upper jaw.
