# Supplementary material for: Structural basis of RNA polymerase inhibition by viral and host factors
Source: Nat Commun. 2021 Sep 17;12:5523. doi: 10.1038/s41467-021-25666-5 (PMC8448823; doi:10.1038/s41467-021-25666-5)
Supplement: Supplementary file 5 — Reporting Summary [file 41467_2021_25666_MOESM5_ESM.pdf]

nature portfolio

Corresponding author(s): Finn Werner

Last updated by author(s): Aug 16, 2021

Reporting Summary

Nature Portfolio wishes to improve the reproducibility of the work that we publish. This form provides structure for consistency and transparency in reporting. For further information on Nature Portfolio policies, see our Editorial Policies and the Editorial Policy Checklist.

Statistics

For all statistical analyses, confirm that the following items are present in the figure legend, table legend, main text, or Methods section.

n/a	Confirmed	
☐	☒	The exact sample size (n) for each experimental group/condition, given as a discrete number and unit of measurement
☐	☒	A statement on whether measurements were taken from distinct samples or whether the same sample was measured repeatedly
☒	☐	The statistical test(s) used AND whether they are one- or two-sided<br>*Only common tests should be described solely by name; describe more complex techniques in the Methods section.*
☒	☐	A description of all covariates tested
☒	☐	A description of any assumptions or corrections, such as tests of normality and adjustment for multiple comparisons
☒	☐	A full description of the statistical parameters including central tendency (e.g. means) or other basic estimates (e.g. regression coefficient) AND variation (e.g. standard deviation) or associated estimates of uncertainty (e.g. confidence intervals)
☒	☐	For null hypothesis testing, the test statistic (e.g. F, t, r) with confidence intervals, effect sizes, degrees of freedom and P value noted<br>*Give P values as exact values whenever suitable.*
☒	☐	For Bayesian analysis, information on the choice of priors and Markov chain Monte Carlo settings
☒	☐	For hierarchical and complex designs, identification of the appropriate level for tests and full reporting of outcomes
☒	☐	Estimates of effect sizes (e.g. Cohen's d, Pearson's r), indicating how they were calculated

Our web collection on statistics for biologists contains articles on many of the points above.

Software and code

Policy information about availability of computer code

Data collection	EPU v1 software was used for EM data collection
Data analysis	RELION v3.0 and cryoSPARC v2 were used for data processing; RELION v3.0 and Phenix v1.15 were used for map sharpening; Phenix v1.19 and Coot v0.8.9.1 were used for model fitting and refinement. Molprobity webserver was used for data validation. Other softwares and webservers: Chimera v1.13 and LigPlot+ v2; Modeller, Espript3, and PROMALS3D webservers.

For manuscripts utilizing custom algorithms or software that are central to the research but not yet described in published literature, software must be made available to editors and reviewers. We strongly encourage code deposition in a community repository (e.g. GitHub). See the Nature Portfolio guidelines for submitting code & software for further information.

Data

Policy information about availability of data

All manuscripts must include a data availability statement. This statement should provide the following information, where applicable:

- Accession codes, unique identifiers, or web links for publicly available datasets
- A description of any restrictions on data availability
- For clinical datasets or third party data, please ensure that the statement adheres to our policy

Structural data generated in the current study are available on the RCSB Protein Data Bank, and the corresponding EM maps deposited on the Electron Microscopy Data Bank. PDB accession codes: apo-RNAP polymerase pdb 7ok0, RNAP-RIP complex 7oq4, and RNAP-TFS4 complex 7oqy. EMDB accession codes: apo-RNAP EMDB-12960, RNAP/RIP complex EMD-13026, and RNAP/TFS4 complex EMDB-13034

March 2021

Field-specific reporting

Please select the one below that is the best fit for your research. If you are not sure, read the appropriate sections before making your selection.

☒ Life sciences ☐ Behavioural & social sciences ☐ Ecological, evolutionary & environmental sciences

For a reference copy of the document with all sections, see nature.com/documents/nr-reporting-summary-flat.pdf

Life sciences study design

All studies must disclose on these points even when the disclosure is negative.

Sample size	Protein concentration throughout this study was measured using the Qubit 2.0 fluorimeter (Invitrogen) and the SyproOrange as detection dye. For EM experiments, the sample size was experimentally determined based on particles distribution on micrographs and example are shown in supplementary figures 1b, 5b and 6b.
Data exclusions	No data were excluded.
Replication	For EM experiments, replications were not carried out other than sample optimizations prior data collection that allowed to identify the correct grids to use. Data from EM are rarely presented as replicates because replication can affect data quality.<br>EMSA assays were replicated successfully four times. In the manuscript, we presented one example.<br>The western blot was repeated twice with successful results.
Randomization	Western blot: randomization was not possible because the cells used for immunodetection needed to be sampled at specific time point.<br>cryo-EM: particles and their orientations are randomly distributed on each micrograph as shown in supplementary figures 1b, 5b, and 6b.<br>EMSA assays: randomization is not possible because the assays is concentration-dependent.
Blinding	Blinding is not relevant because there are no animals or patients involved in the study.

Reporting for specific materials, systems and methods

We require information from authors about some types of materials, experimental systems and methods used in many studies. Here, indicate whether each material, system or method listed is relevant to your study. If you are not sure if a list item applies to your research, read the appropriate section before selecting a response.

Materials & experimental systems

n/a	Involved in the study	
☐	☒	Antibodies
☒	☐	Eukaryotic cell lines
☒	☐	Palaeontology and archaeology
☒	☐	Animals and other organisms
☒	☐	Human research participants
☒	☐	Clinical data
☒	☐	Dual use research of concern

Methods

n/a	Involved in the study	
☒	☐	ChIP-seq
☒	☐	Flow cytometry
☒	☐	MRI-based neuroimaging

Antibodies

Antibodies used	Primary antibodies: rabbit polyclonal anti-ATV RIP (Davids Biotechnology) used at 1:1,000 dilution and sheep polyclonal anti-Sso Alba (kindly provided by Malcom White, University of St. Andrews, UK) used at 1:3,000 dilution. In both cases the antibodies were not commercially available but custom-prepared by arising the antibodies against recombinantly expressed protein samples.<br>Secondary antibodies: donkey anti-sheep IgG conjugated with Dylight 488 used at 1:2,000 dilution and donkey anti-rabbit IgG conjugated with Dylight 680 used at 1:10,000. Both antibodies were purchased from Bethyl Laboratories (UK) with catalog codes A130-100D6 and A120-208D6, respectively.
Validation	ATV RIP antibodies were purified from serum on Protein A resin. Validation and final working dilution were experimentally determined by testing different antibody dilutions in wester blot on recombinantly expressed RIP.<br>All other antibodies were used according to manufacter's protocols.<br>All antibodies were used in the immunodetection experiment as reported on Methods, Immunotetection paragraph, and results shown in Figure 3.
