# Supplementary material for: Structural basis of RNA polymerase inhibition by viral and host factors
Source: Nat Commun. 2021 Sep 17;12:5523. doi: 10.1038/s41467-021-25666-5 (PMC8448823; doi:10.1038/s41467-021-25666-5)
Supplement: Supplementary file 6 — Source Data [file 41467_2021_25666_MOESM6_ESM.zip › SORCE DATA description.docx]

**SORCE DATA**

**EMSA assay.** Uncropped native gel used in figure 3d. The detected fluorescence signal of the Cy3-labelled DNA and DNA/RNA scaffolds is shown on the left, and the corresponding ^32^P-labelled RIP signal on the right. The (+) signs indicate 0.2 µM Saci RNA polymerase, and 0.4 µM RIP. Dose-response titration includes increasing concentrations of RIP (0.2; 1; 2 µM) or DNA/RNA scaffold (0.1; 0.5; 1 µM).

**Western blot analysis.** Uncropped western blot membrane shown in figure 3e. RIP was detected using a secondary antibody conjugated with Dylight 680 which is shown here in green, whereas the control Alba was detected with a secondary antibody conjugated with Dylight 488 in red. Samples were taken at specific time intervals to follow the virus cell cycle as reported in the figure.

**SDS-PAGE of cryo-EM samples.** Uncropped SDS-PAGE of the purified RNAP, RIP, and TFS4 samples used for the cryo-EM data collection shown in Supplementary figures 1a, 5a, and 6a. Each sample was loaded twice in two different concentrations.
