# Supplementary figures and images for: Structural basis of RNA polymerase inhibition by viral and host factors
Source: Nat Commun. 2021 Sep 17;12:5523. doi: 10.1038/s41467-021-25666-5 (PMC8448823; doi:10.1038/s41467-021-25666-5)

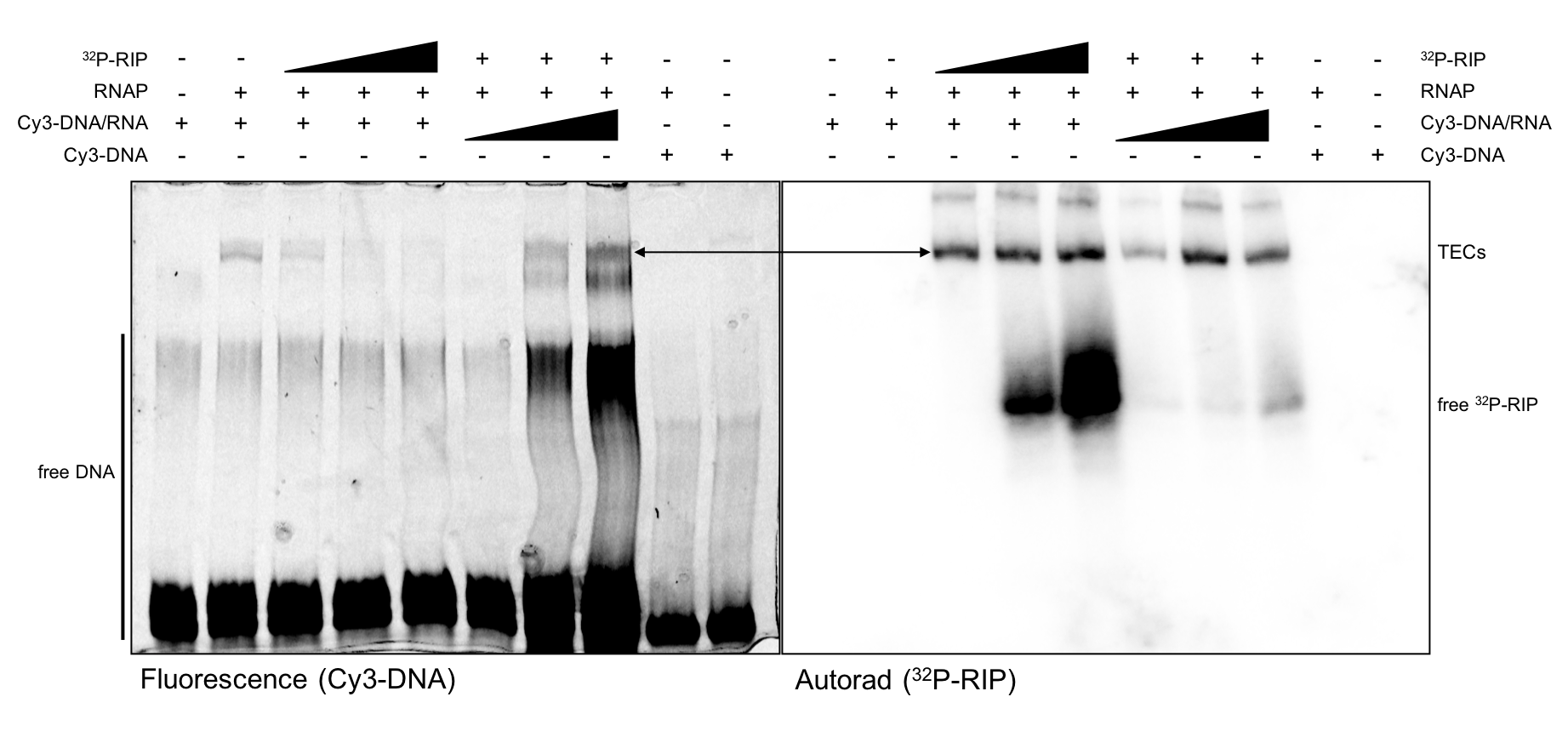

Supplement: Supplementary file 6 — Source Data [file 41467_2021_25666_MOESM6_ESM.zip › EMSA assay.png]

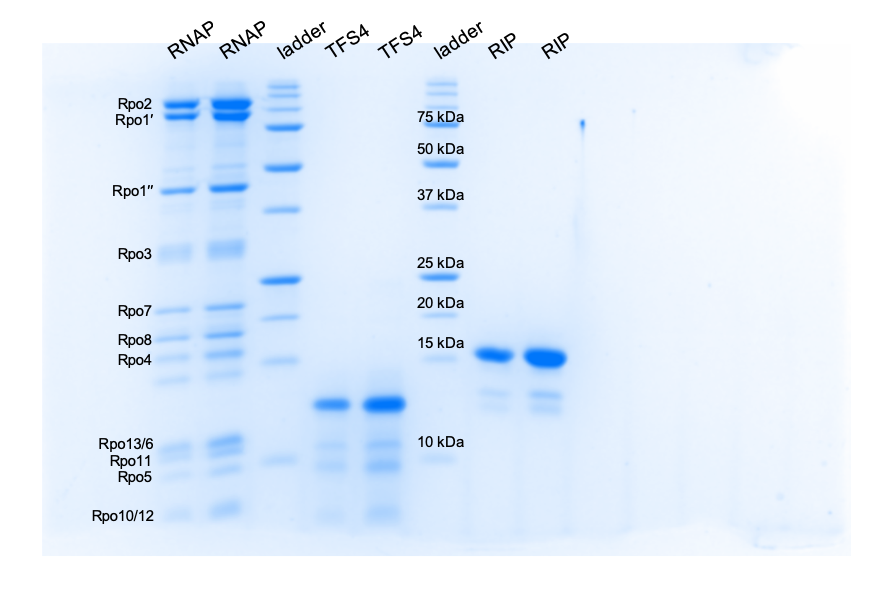

Supplement: Supplementary file 6 — Source Data [file 41467_2021_25666_MOESM6_ESM.zip › SDS-PAGE of cryo-EM sample.png]

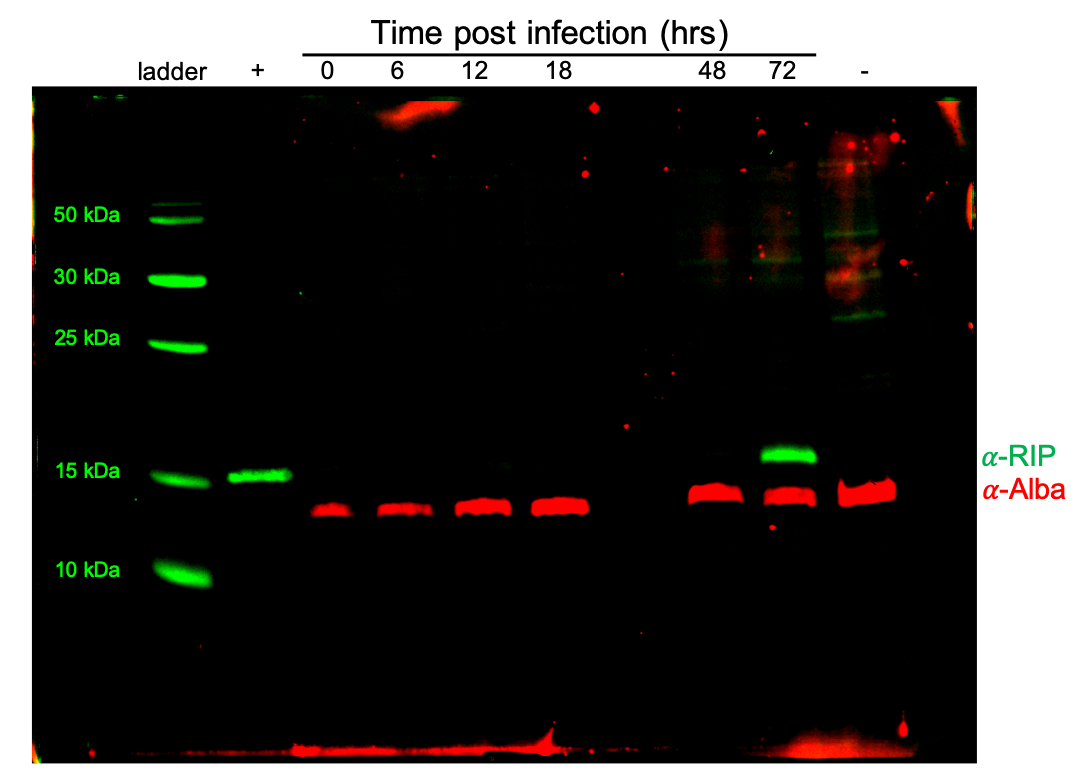

Supplement: Supplementary file 6 — Source Data [file 41467_2021_25666_MOESM6_ESM.zip › Western blot analysis.png]
